# Case Report: Concurrent retinal vasculitis and optic neuritis in systemic lupus erythematosus

**DOI:** 10.3389/fimmu.2025.1646850

**Published:** 2025-08-18

**Authors:** Di Jin, Xun Liu, Yuwei Wang, Longjiang Fang, Weiduo Nie, Chen Li, Sheng-Guang Li, Ming Li

**Affiliations:** ^1^ Department of Rheumatology, Weifang People’s Hospital, Weifang, Shandong, China; ^2^ Department of Ophthalmology, Weifang People’s Hospital, Weifang, Shandong, China; ^3^ Department of Emergency Internal Medicine, Weifang People’s Hospital, Weifang, Shandong, China; ^4^ Department of Radiology, Weifang People’s Hospital, Weifang, Shandong, China; ^5^ School of Traditional Chinese Medicine, Beijing University of Chinese Medicine, Beijing, China; ^6^ Department of Dermatology, Tianjin Institute of Integrative Dermatology, Tianjin Academy of Traditional Chinese Medicine Affiliated Hospital, Tianjin, China; ^7^ Department of Rheumatology and Immunology, Peking University International Hospital, Beijing, China

**Keywords:** systemic lupus erythematosus, retinal vasculitis, optic neuritis, autoimmune ocular disease, immunosuppressive therapy

## Abstract

Systemic lupus erythematosus (SLE) is a multisystem autoimmune disease that can affect the ocular system, with retinal vasculitis and optic neuritis being rare but serious manifestations. We present a case of a 26-year-old female with newly diagnosed SLE who developed both retinal vasculitis and optic neuritis, leading to progressive visual impairment. She was successfully treated with methylprednisolone and rituximab, achieving significant visual recovery. A review of existing literature highlights the diagnostic challenges, pathophysiology, and optimal treatment strategies for such cases. Our findings emphasize the importance of early recognition and aggressive immunosuppressive therapy in improving patient outcomes.

## Introduction

Systemic lupus erythematosus (SLE) is a chronic autoimmune disorder characterized by multisystem involvement, including renal, neurological, cardiovascular, and ocular manifestations. Among its ocular complications, lupus retinopathy is the most common. Lupus retinopathy, referring specifically to retinal hemorrhages, cotton-wool spots, retinal edema, and vascular occlusions directly caused by SLE (excluding drug-induced forms such as chloroquine-induced retinopathy), occurs in approximately 10% to 29% of SLE patients (1). However, retinal vasculitis and optic neuritis remain rare but severe manifestations, often leading to significant visual impairment if left untreated (2). These complications typically arise in the context of high systemic disease activity and are associated with vascular inflammation, immune complex deposition, and autoantibody-mediated endothelial dysfunction (3, 4).

Retinal vasculitis in SLE is often characterized by perivascular inflammation, vascular leakage, and potential occlusion, leading to ischemic retinal damage and neovascularization (3, 5). Optic neuritis, on the other hand, results from inflammation of the optic nerve, leading to acute vision loss, dyschromatopsia, and relative afferent pupillary defects (6). These conditions pose significant diagnostic challenges, as their presentations can overlap with other autoimmune, infectious, and thrombotic disorders.

Early recognition and appropriate immunosuppressive treatment are crucial for preserving vision and preventing irreversible damage. Corticosteroids remain the mainstay of initial therapy, but refractory or severe cases may benefit from biologic therapies such as rituximab (7). In this report, we present a case of a young female with newly diagnosed SLE who developed concurrent retinal vasculitis and optic neuritis, successfully treated with corticosteroids and rituximab. A literature review is included to provide further insight into the epidemiology, pathophysiology, diagnosis, and treatment options for these rare but devastating ocular manifestations of SLE.

## Case presentation

A 26-year-old woman presented with a one-month history of facial erythema and a 10-day history of progressive blurred vision. Initially, she noticed a symmetrical erythematous rash over her face but did not seek medical attention. Ten days prior to admission, she developed sudden bilateral vision loss without identifiable triggers. She reported no fever, chills, limb numbness, hair loss, dry eyes, dry mouth, Raynaud’s phenomenon, muscle pain or weakness, joint stiffness, or systemic symptoms such as abdominal pain or diarrhea.

She was evaluated at the local ophthalmic hospital and diagnosed with optic neuritis. She received three doses of intravenous methylprednisolone (500 mg/day) with minimal improvement in visual acuity. Two days later, she experienced intermittent headaches, palpitations, nausea, and vomiting, with systolic blood pressure peaking at 170 mmHg. These symptoms prompted her admission to our hospital for further evaluation.

**Figure 1:**
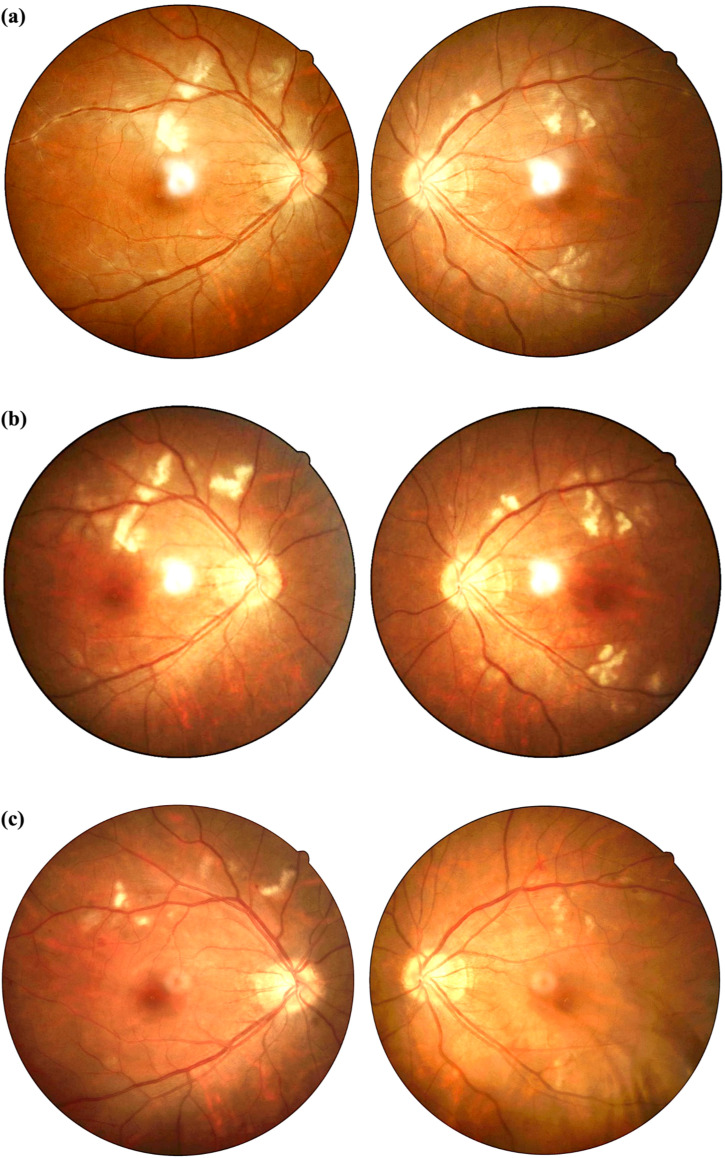
**(a-c)** Comparison of fundus photographs before and after treatment. **(a)** Fundus photographs obtained on admission. Bilateral optic disc edema with blurred margins. Posterior pole retinal white exudates. Right eye demonstrates enhanced superior temporal vascular reflex and hemorrhagic spots. **(b)** Fundus photographs obtained on 12 days after admission. Persistent bilateral disc edema and retinal exudates. **(c)** Fundus photographs at three-month post-treatment follow-up. Marked reduction of exudates in both eyes, with residual disc margin blurring. Compared with before treatment, the patient’s retinal vasculitis lesions showed significant improvement after treatment.

**Figure 2:**
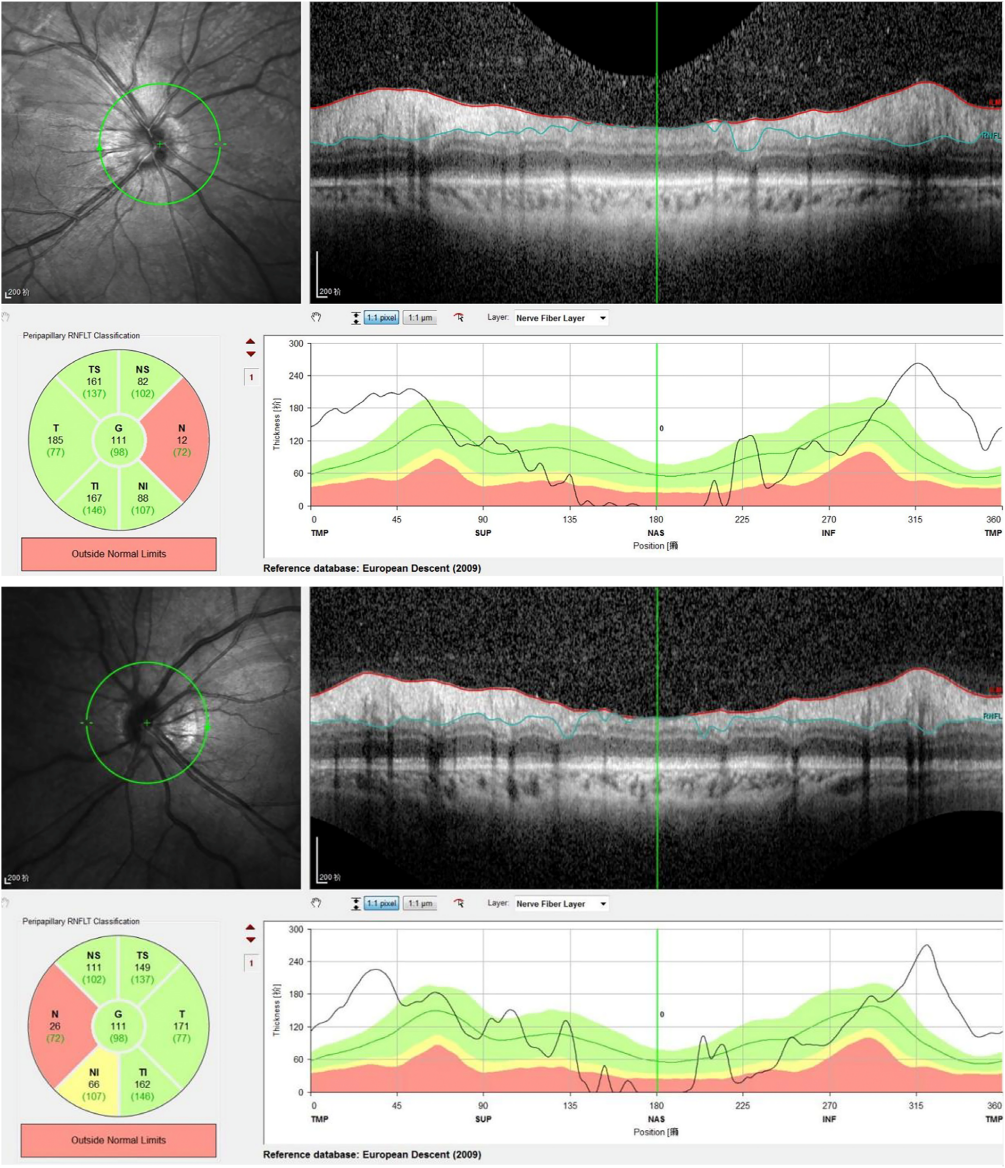
Bilateral retinal nerve fiber layer (RNFL) OCT scan reveals: thinning of nasal RNFL thickness and thickening of temporal RNFL thickness, indicating bilateral optic neuritis-induced RNFL damage. In this patient, the nasal RNFL is significantly thinned, being the first to be affected and suffering the most severe damage. The relative thickening of the temporal RNFL may be attributed to inflammation-induced axonal swelling and optic disc edema, leading to increased RNFL thickness.

On admission, her best corrected visual acuity was 0.6 in the right eye and 0.8 in the left eye. Fundoscopic examination revealed bilateral optic disc edema with soft exudates, findings consistent with concurrent retinal vasculitis (**Figure 1a**). Enhanced vascular reflex is noted in the superior temporal retinal vessels, with scattered hemorrhagic spots. Scattered white exudates are observed in the posterior pole of the retina. Bilateral OCT scans of the optic nerve fiber layer (RNFL) thickness on admission reveal thinning of the nasal retinal nerve fiber layer and thickening of the temporal retinal nerve fiber layer (**Figure 2**). Her blood pressure was elevated, and physical examination showed a butterfly rash across her face. However, there was no alopecia, joint swelling, or other cutaneous abnormalities.

**Figure 3:**
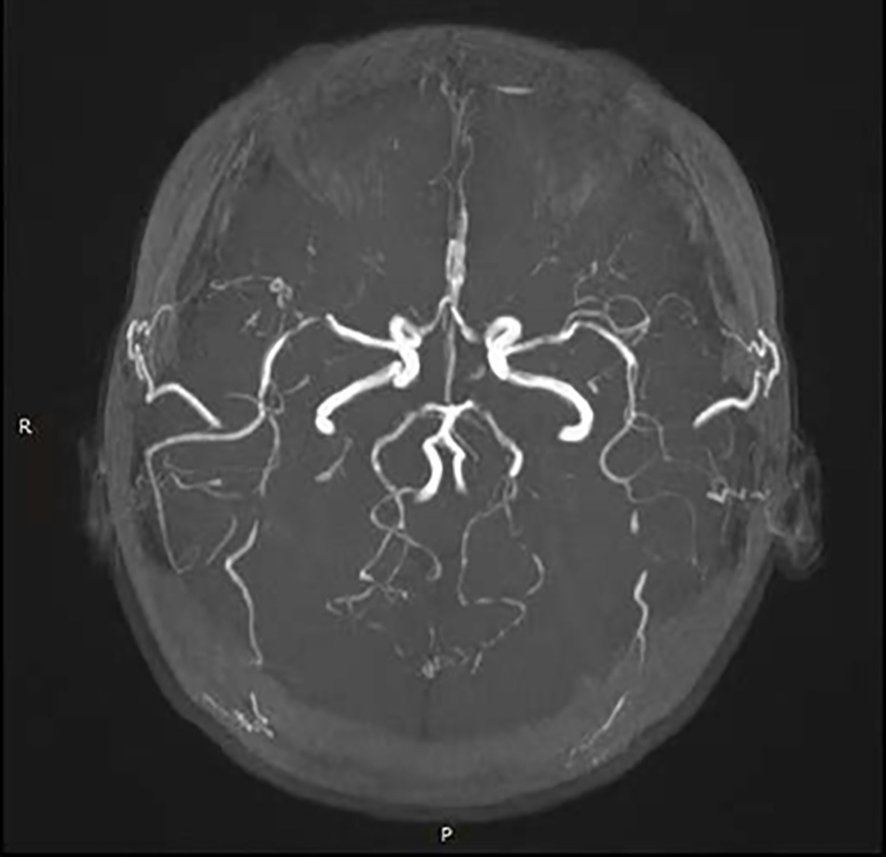
Cranial MRA demonstrated vascular abnormalities consistent with secondary cerebral vasculitis. Cranial MRA demonstrated patent bilateral anterior cerebral arteries, middle cerebral arteries, and posterior cerebral arteries with diminished distal branching, diffuse luminal caliber irregularities, and multiple foci of severe stenosis.

Laboratory investigations revealed leukopenia with a white blood cell count of 1.51 × 10^9^/L, while routine liver and kidney function tests were normal. Inflammatory markers showed a serum amyloid A level of 12.45 mg/L and an erythrocyte sedimentation rate (ESR) of 23 mm/h. Autoantibody testing revealed a nuclear homogeneous pattern on ANA with a titer of 1:3200 and strongly positive anti-dsDNA antibodies at 188.73 IU/mL. Additional positive findings included antibodies against ds-DNA, nucleosome, histone, U1-snRNP, SS-A/Ro52, SS-A/Ro60, SS-B/La, and AMA-M2. Complement levels were significantly reduced, with C3 at 0.46 g/L and C4 at 0.07 g/L. Immunoglobulin G was elevated at 18.00 g/L. Other tests, including antineutrophil cytoplasmic antibodies (ANCA), lupus anticoagulant, antiphospholipid antibodies, anticardiolipin antibodies, C-reactive protein, ferritin, and 24-hour urine protein quantification, were within normal limits. The patient tested negative for infectious diseases such as tuberculosis and hepatitis B. Imaging studies further supported the diagnosis. Cranial magnetic resonance angiography (MRA) demonstrated vascular abnormalities consistent with secondary cerebral vasculitis (**Figure 3**). Ultrasonography of the heart, abdomen, and other systemic evaluations revealed no abnormalities. Transient hypertension observed was likely due to acute inflammatory response and corticosteroid administration. Renal function tests and proteinuria assessments were normal, ruling out active lupus nephritis. Cranial imaging (MRI/MRA) excluded posterior reversible encephalopathy syndrome (PRES). Due to the patient’s urgent admission and personal preference, fluorescein angiography and visual evoked potentials (VEP) were not performed initially. However, optic nerve OCT and cranial MRI/MRA clearly documented optic nerve inflammation and cerebral vasculitis.

Based on her clinical presentation, ophthalmic findings, strongly positive anti-dsDNA and other autoantibodies, reduced complement levels, and systemic symptoms, a diagnosis of SLE was made. The ocular findings of optic neuritis and retinal vasculitis were identified as severe complications of SLE. Differential diagnoses such as infectious etiologies (tuberculosis, syphilis), autoimmune disorders (granulomatosis with polyangiitis, neuromyelitis optica), and hypertensive retinopathy were considered and systematically excluded through laboratory tests and imaging. The patient was treated with intravenous methylprednisolone (80 mg/d), followed by two doses of intravenous Rituximab (500 mg on the 2nd day post-admission and two weeks after admission) to control systemic inflammation and immunologic activity. Significant improvement was noted: her visual acuity returned to 1.0 bilaterally, and the facial erythema resolved before discharge. Fundus photographs obtained on 12 days after admission. Persistent bilateral disc edema and retinal exudates (**Figure 1b**). Hydroxychloroquine initiation was temporarily deferred due to severe ocular involvement. Ophthalmology consultation advised postponing it to avoid confounding retinal assessments. Initiation is planned after confirming retinal stability at 6 months. A lower initial dose of mycophenolate mofetil (750 mg/day) was chosen due to the patient’s low body weight and gastrointestinal sensitivity, with gradual escalation to 1500 mg/day after three months.

The patient was discharged on the 15th day after admission. She was prescribed oral prednisolone (40 mg/d) and mycophenolate mofetil (750 mg/d) for maintenance therapy. Antihypertensive medications were briefly administered during hospitalization but discontinued prior to discharge. At three months post-discharge, the patient maintained clinical stability with successful corticosteroid tapering and absence of systemic disease activity. Blood pressure remained stable within normal range throughout the follow-up period. Follow-up fundoscopic examination demonstrated significant improvement in retinal findings. Scattered white exudates are observed in the posterior pole of the retina, with a significant reduction in exudates compared to previous examinations. (**Figure 1c**). Laboratory test indicators and disease activity show significant improvement compared with before treatment (**Table 1**).

**Table 1:** The laboratory results and SLEDAI scores at baseline and after three months of therapy.

Parameter	Baseline	After 3 months	Reference range
Anti-dsDNA (IU/mL)	188.73	9.90	<24
Complement C3 (g/L)	0.46	1.01	0.7-1.4
Complement C4 (g/L)	0.07	0.31	0.1-0.4
IgG (g/L)	18.00	10.20	8.6-17.4
White Blood Cell (×10^9^/L)	1.51	6.35	3.5-9.5
ESR (mm/h)	23	8	<20
SLEDAI score	16	0	

## Discussion

This case underscores the importance of early recognition and prompt immunosuppressive therapy in SLE-related ocular vasculitis and optic neuritis, particularly in severe cases requiring biologic intervention. The concurrence of retinal vasculitis and optic neuritis in SLE presents a diagnostic challenge due to overlapping clinical features with other autoimmune and infectious etiologies. The patient initially presented with ocular manifestations as the first clinical sign of SLE, significantly complicating both diagnosis and therapeutic management. To contextualize the findings, we compared the present case with 30 previously published cases of SLE-related severe ocular manifestations (**Tables 2**, **3**). The dataset categorized these cases into three groups: SLE with both retinal vasculitis and optic neuritis (7 cases) (8–14), SLE with retinal vasculitis alone (10 cases) (7, 15–23), and SLE with optic neuritis alone (13 cases) (6, 24–33). Our case falls into the first category, which represents the most severe ocular involvement.

**Table 2:** The common treatments, overall prognosis and typical follow-up for retinal vasculitis and optic neuritis in SLE.

Group	Cases (n)	Common treatments	Main treatment effect	Overall prognosis	Typical follow-up
Retinal Vasculitis Only	10	IV steroids, Rituximab, Cyclophosphamide, photocoagulation	Some vision improvement	Moderate	12 months
Optic Neuritis Only	13	Prednisone	Partial vision recovery	Progressive optic atrophy	6 months
Retinal Vasculitis & Optic Neuritis	7	IV steroids, Cyclophosphamide, Rituximab	Limited or unclear improvement	Poor	Not specified

**Table 3:** Clinical characteristics of SLE patients with retinal vasculitis and optic neuritis: A comparative overview.

Author(s) / References	Gender	Age	Eye disease/diagnosis	Fundoscopy/Eye imaging results	Other diagnosis	Time of onset of eye symptoms before treatment	Main treatment measures	Effect	Prognosis	Follow-up time
Mavrikakis-1983 / (8)	Female	34	Retinal vasculitis, optic neuritis	Fluorescein angiography showed small narrow disc vessels and hypofluorescence of the disc appeared in the arterial and arteriovenous phase. In the late phase a trace of hyperfluorescence was observed. The retinal vessels showed diffuse retinal vasculitis, more intensive in the left eye.	Jaccoud's syndrome	Not mentioned	Not mentioned	Not mentioned	Not mentioned	Not mentioned
Read RW-2000 / (9)	Female	31	Retinal vasculitis, optic neuritis	Retinal infarctions, hemorrhages, optic neuropathy	None	Rapid onset	IV steroids, Cyclophosphamide, Panretinal photocoagulation	Vision loss to 3/200 in one eye	Poor	Not specified
Barkeh HJ-2002 / (10)	Female	19	Retinal vasculitis, optic neuritis	Hyperemic, swollen optic disc, periphlebitis, exudative macular detachment	None	4 days	IV methylprednisolone, oral prednisolone taper	Vision improved to 6/18	Good	8 months
Papadaki TG-2006 / (11)	Female	31	Retinal vasculitis, optic neuritis	Arterial sheathing, hemorrhages, capillary non-perfusion	None	Not specified	IV Cyclophosphamide, Panretinal photocoagulation, Vitrectomy	Vision deteriorated?	Poor	7 months
Donnithorne KJ-2013 / (12)	Female	16	Retinal vasculitis with ischemic optic neuropathy	Cotton-wool spots, neovascularization, optic neuropathy	None	1 year	IV steroids, Rituximab, Cyclophosphamide, Panretinal photocoagulation	Some vision improvement	Poor	12 months
Dhirani N-2017 / (13)	Female	34	Retinal vasculitis, optic neuritis	Diffuse retinal hemorrhage, pallid optic nerve swelling, diffusely swollen macula with a cherry-red appearance, and vascular sheathing in fundus examination in the right eye	None	3 days	Plasmapheresis, Rituximab	Improved to 20/30 vision	Stable	12 months
Chin D-2021 / (14)	Female	29	Retinal vasculitis, optic neuritis	Retinal hemorrhages, capillary non-perfusion, optic nerve ischemia	Mixed Connective Tissue Disease	2 days	IV steroids, Rituximab, Cyclophosphamide, laser photocoagulation	Partial vision recovery	Moderate	Several months
Koch JW-1992 / (15)	Female	37	Retinal vasculitis (Occlusive)	Widespread ischemia, vascular occlusion, neovascularization	None	12 years	Maximal immunosuppression, Panretinal photocoagulation, Cryotherapy	Progressive vision loss	Poor	6 months
Hickman RA-2010 / (16)	Female	33	Retinal vasculitis bilateral	Widespread hemorrhages, cotton-wool spots, flame hemorrhages	None	1 week	IV steroids, Rituximab, Cyclophosphamide	Resolution of vasculitis, limited vision recovery	Moderate	12 months
Monov S-2017 / (17)	Female	25	Retinal vasculitis (Necrotizing )	Fundus photograph showed cotton-wool spots, branch artery occlusion, hemorrhages	APS	1 week	IV steroids, Immunoglobulin, Cyclophosphamide, Azathioprine	Partial visual recovery	Moderate	10 months
Butendieck RR-2012 / (18)	Female	38	Retinal vasculitis	Widespread hemorrhages, periphlebitis, ischemic macular thickening	None	2 days	IV steroids, cyclophosphamide, mycophenolate mofetil	Vision improved to 20/30	Favorable	5 months
Tselios K-2017 / (19)	Female	38	Retinal vasculitis	Vitreous hemorrhage, optic disc neovascularization	None	Rapid onset	IV steroids, Rituximab, Cyclophosphamide, Panretinal photocoagulation	Vision improvement, no relapse	Good	6 months
Luo Y-2018 / (20)	Male	37	Retinal vasculitis	Cotton-wool spots, hemorrhages	MAS, APS	Several weeks	IV steroids, Mycophenolate Mofetil	Vision stabilized	Moderate	12 weeks
Alhassan E-2021 / (21)	Female	14	Retinal vasculitis Bilateral	Diffuse hemorrhages, white retinal lesions, blurred optic disc margins	Schizophrenia	4 days	IV steroids, hydroxychloroquine, azathioprine	Significant visual improvement	Good	12 months
Kuthyar S-2022 / (22)	Female	30	Retinal vasculitis	Ischemic vein occlusion, macular edema, vascular leakage	None	Not specified	IV steroids, Mycophenolate Mofetil, Adalimumab	Resolution of vasculitis	Favorable	27 months
Aldhefeery N-2023 / (23)	Male	34	Retinal vasculitis Bilateral	Cotton-wool spots, hemorrhages, macular edema, vascular beading	APS	3 weeks	IV steroids, oral prednisone taper	Vision improved to 20/20	Favorable	18 months
Matijaševic MI-2023 / (7)	Not specified	Not specified	Retinal vasculitis	Retinal hemorrhages, diffuse vasculitis (fundoscopy, angiography)	MAS	Not specified	Rituximab, intravitreal Bevacizumab, laser photocoagulation	Significant improvement in visual acuity	Favorable prognosis	12 months
Hackett-1974, Case 1 / (24)	Female	11	Optic neuritis	minimal disk edema, evolving in a few days into severe disk swelling with flame hemorrhages, congested veins, and retinal edema was found in Funduscopy examination	Myasthenia gravis	less than two weeks	Prednisone	Partial recovery in vision	Progressive optic atrophy	12 months
Hackett-1974, Case 2 / (24)	Female	23	Optic neuritis	slight edema and increased vascularity of the optic disk and engorgement of the retinal vessels was found in Funduscopy examination, with no changes in the retina.	transverse myelitis syndrome, neuromyelitis optica	Not mentioned	Prednisone	Partial recovery in vision within several weeks, then lost all visual function in the left eye	Progressive optic atrophy	36 months
Hackett-1974, Case 3 / (24)	Female	27	Optic neuritis	Not done	transverse myelitis syndrome	Not mentioned	Prednisone	No visual recovery	Severe optic atrophy	6 months
Oppenheimer S-1986 / (25)	Female	47	Optic neuritis	Optic disc pale and ischemic	Myelopathy	Within one week	Steroids, cyclophosphamide	Partial improvement, and recurrent when steroids tapered to 5 mg/d	Chronic central scotoma	48 months
Kenik JG-1987 / (26)	Female	27	Optic neuritis	Funduscopy examination was normal	Cerebral infarction, transverse myelitis	within Two weeks	Methylprednisolone, Cyclophosphamide	Improved vision, persisted motor deficits	motor deficits persisted without improvement, vision almost total recovery	2 months
Im CY-2002 / (27)	Female	21	Optic neuritis bilateral, Central retinal vein occlusion	Bilateral nerve-fiber layer infarcts, intraretinal hemorrhage, mild hyperemia, blurred disc margins; Fluorescein angiography showed dye leakage around optic disc, tortuosity of retinal veins, blockage of background fluorescence due to hemorrhage	APS	2 days	Hemodialysis, Blood Transfusion, High-dose steroids (IV methylprednisolone), Oral corticosteroids, Cyclophosphamide	Right eye improved to 120/200 vision, left eye remained at counting fingers level; Cotton-wool spots and hemorrhages persisted	Right eye partially recovered, left eye retained large central scotoma	2 months
Birnbaum J-2008 / (28)	Female	38	Optic neuritis	Not reported	recurrent myelitis, bilateral sensorineural hearing loss	Not specified	Rituximab, Cyclophosphamide	Improved, no further attacks	Stable	12 months
Lin YC-2009 / (29)	Female	28	Optic neuritis bilateral	MRI showed segmental enhancement of optic nerves	None	Not specified	Steroid pulse therapy	Poor response in third attack, optic atrophy developed	Poor in later attacks	Lost to follow-up after final recorded VA
Pellkofer H-2010 / (30)	Female	44	Optic neuritis	Brain MRI showed white matter lesions	Myelitis	Not specified	Rituximab, cyclophosphamide	Reduced relapse frequency	Poor without aggressive treatment	102 months
Patra S-2011 / (31)	Female	11	Optic Neuritis bilateral	Bilateral disc edema (optic nerve ultrasound)	APS, Evan’s Syndrome	Rapid deterioration	IV methylprednisolone, cyclophosphamide pulses, anticoagulants	No perception of light, progressed to optic atrophy	Poor visual outcome	6 months
Srimanan W-2022 / (32)	Female	11	Optic neuritis bilateral	Severe bilateral disc edema, peripapillary hemorrhage	Intracranial hypertension	2 weeks	IV Methylprednisolone	Improved vision to 20/50	Moderate recovery	5 months
Prakash S-2023 / (6)	Female	22	Optic neuritis	Cherry-red spot, tomato splash background, tortuous veins, hyperemic disc in LE	APS	Not specified	IV steroids, anticoagulants	Vision restored in LE, lost in RE	Good for LE, poor for RE	6 months
Kang M-2023 / (33)	Female	42	Optic neuritis	Not mentioned	None	Not specified	IV Methylprednisolone	Improved vision to 20/25	Stable after vaccine-related flare-up	1 month
Present case	Female	26	Retinal vasculitis, optic neuritis	Fundoscopic examination revealed bilateral optic disc edema with soft exudates	None	Within two weeks	Steroids, Rituximab	Remission achieved, maintained vision	Controlled systemically	3 month

The present case exhibited highly active systemic lupus, with markedly elevated ds-DNA titers, low complement levels, and concurrent neurovascular involvement, as suggested by MRA findings. This aligns with trends observed in patients with both retinal vasculitis and optic neuritis, who frequently exhibited multisystem disease, including lupus nephritis and CNS lupus (20, 34) In contrast, patients with retinal vasculitis alone also had active SLE but tended to have fewer concurrent neuropsychiatric symptoms (19, 22). Retinal vasculitis in these cases was often the first sign of systemic lupus exacerbation. Patients with isolated optic neuritis had a more variable degree of systemic lupus activity. Some had isolated optic neuritis with minimal systemic manifestations, while others developed CNS lupus over time (35).

In terms of treatment, our patient received intravenous methylprednisolone followed by rituximab and mycophenolate mofetil, achieving significant visual recovery. This aligns with treatment approaches observed in previous reports of concurrent retinal vasculitis and optic neuritis in SLE, where corticosteroid monotherapy was typically inadequate, necessitating additional immunosuppressive agents such as cyclophosphamide or rituximab to effectively control disease activity and preserve vision (12–14).

Notably, therapeutic responses and prognoses differ substantially between lupus-associated retinal vasculitis and optic neuritis. Patients with isolated retinal vasculitis generally show good initial responses to corticosteroids alone; however, maintaining remission frequently requires additional long-term immunosuppression (e.g., azathioprine or mycophenolate mofetil) (15, 19, 22). Early and aggressive intervention usually results in favorable outcomes, although visual prognosis can vary significantly depending on the extent and rapidity of vascular occlusion. Those with limited vaso-occlusion often achieve better visual prognoses, whereas cases involving severe ischemic retinopathy may lead to permanent vision loss despite therapy (15, 16).

In contrast, patients presenting with lupus-associated optic neuritis tend to respond initially to high-dose corticosteroids, often with noticeable improvement in acute visual symptoms. However, unlike typical demyelinating optic neuritis (as seen in multiple sclerosis), visual recovery in lupus-related optic neuritis is frequently incomplete. Long-term outcomes tend to be less favorable, marked by partial visual recovery, progressive optic nerve atrophy, or recurrent episodes despite continued immunosuppressive therapy (6, 29–31, 33). Approximately one-third of these patients experience relapses, underscoring the difficulty of achieving sustained remission (30, 31).

Patients like ours, presenting concurrently with both retinal vasculitis and optic neuritis, generally experience the most severe ocular involvement and thus have the worst prognoses overall. Many previously reported cases resulted in irreversible visual impairment due to profound retinal ischemia or persistent optic nerve damage (9, 11, 12). However, our patient achieved remarkable visual recovery, making her one of the best responders within this severe subgroup. Nonetheless, the high risk of disease recurrence mandates ongoing, carefully tailored immunosuppressive management. Therefore, recognizing these distinct therapeutic responses and prognostic outcomes is critical for rheumatologists, ophthalmologists, and neurologists involved in managing ocular manifestations of SLE. Early, aggressive, and individualized immunosuppressive therapy—along with diligent monitoring—is essential to optimize long-term visual outcomes in these challenging cases.

## Conclusion

This case highlights the need for rapid diagnosis and aggressive immunosuppression in severe SLE-related ocular disease. Given that retinal vasculitis often signals active systemic lupus, early recognition is crucial. The association with antiphospholipid antibodies suggests that anticoagulation may be beneficial in select cases to prevent further vaso-occlusive events (6, 27, 31). Additionally, biologic therapies such as rituximab is emerging as promising treatments for refractory disease (7, 22).

Moving forward, continued research is needed to establish standardized treatment protocols for these rare but vision-threatening complications. Our findings reinforce the value of a multidisciplinary approach, integrating rheumatologists, ophthalmologists, and neurologists to achieve optimal patient outcomes. This case contributes to the growing body of literature on SLE-related ocular disease, providing valuable comparative insights between a real-world case and previously documented cases. Future studies with larger cohorts and long-term follow-up are necessary to refine therapeutic strategies and improve patient prognosis.

## Data Availability

The original contributions presented in the study are included in the article/supplementary material. Further inquiries can be directed to the corresponding authors.
